# Automation and artificial intelligence in the clinical microbiology laboratory

**DOI:** 10.3389/fcimb.2026.2000316

**Published:** 2026-10-20

**Authors:** Yaodong Wang, Yongxia Zhang, Na Li, Jinchun He

**Affiliations:** 1 Laboratory Medicine Center, The Second Hospital & Clinical Medical School, Lanzhou University, Lanzhou, Gansu, China; 2 Department of Clinical Laboratory, Yumen City General Hospital (Yumen City First People’s Hospital), Yumen, Gansu, China; 3 Department of General Surgery, The Second Hospital & Clinical Medical School, Lanzhou University, Lanzhou, Gansu, China; 4 Department of Laboratory Medicine, The First Hospital of Lanzhou University, The First School of Clinical Medicine of Lanzhou University, Lanzhou, Gansu, China

**Keywords:** antimicrobial stewardship, artificial intelligence, clinical microbiology, machine learning, total laboratory automation, turnaround time

## Abstract

**Background:**

Clinical microbiology laboratories face persistent pressure to shorten turnaround times, reduce manual workloads, and standardize diagnostic workflows. Total laboratory automation (TLA) and artificial intelligence (AI) are complementary responses to these demands. In this review, we examine how the evidence base for automation and AI in clinical microbiology is distributed along a maturity gradient and what this distribution implies for the next phase of implementation.

**Content:**

Available evidence indicates that automated platforms shorten culture turnaround times and reduce manual workload in observational and multi-center studies, although the scale of benefit depends on specimen type and workflow configuration. Automated reading, identification, and susceptibility testing systems showed high concordance with manual standards for common specimen types, with error rates below commonly cited acceptability thresholds. Evidence for AI performance in plate reading and resistance prediction is abundant; however, most models remain in an assistive phase without prospective validation. Randomized trials of rapid testing and automation rarely demonstrate overall clinical superiority, and patient-oriented benefits remain limited and inconsistent. Guidelines provide directional support, whereas interoperability, quality control, and regulatory frameworks are still under development.

**Summary:**

The priority of the field is not additional single-center performance validation. The next phase requires multi-center prospective verification, interpretability, standardization, and comparative evidence with clinical outcomes as endpoints. AI-assisted evidence tools may accelerate evidence generation, although their utility remains preliminary.

## 1 Introduction

Clinical microbiology laboratories generate microbiological information that supports diagnosis, treatment selection, and infection surveillance (Mencacci et al., 2023). Healthcare-associated infections (HAIs) remain a substantial burden, and microbiology diagnostics are central to their detection, epidemiological understanding, and management (Asokan et al., 2026a). Molecular diagnostic methods have expanded the options for rapid pathogen detection, but conventional workflows still rely on manual inoculation, visual inspection of cultures, and labor-intensive identification and susceptibility testing (Asokan et al., 2026b). However, these processes are time-consuming, show limited standardization, and require considerable technical staff (Antonios et al., 2021). Moreover, a long turnaround time (TAT) delays appropriate antimicrobial therapy, and variability in manual interpretation raises concerns about diagnostic consistency.

Total laboratory automation (TLA) has entered clinical microbiology in response to these bottlenecks. Automated instruments are now capable of handling inoculation, incubation, digital imaging and result documentation throughout the entire analytical workflow (Cherkaoui et al., 2026). In parallel, artificial intelligence (AI) methods are applied to colony recognition, image interpretation, and prediction of antimicrobial resistance (AMR) (Mairi et al., 2025). Machine learning (ML) is also increasingly embedded in laboratory software from automated plate reading to decision support in antimicrobial stewardship (AMS) (Lboukili et al., 2026).

Nevertheless, there are still some issues associated with the current TLA process. As recent reviews have summarized, evidence supporting these technologies is unevenly distributed (Antonios et al., 2021). For example, efficiency gains from automation are documented mainly in observational before-after studies and multi-center audits. First, AI performance is frequently reported on institution-built datasets, without prospective external validation. Second, randomized trials that link faster diagnostics to clinical outcomes remain scarce and several returned negative or mixed results. Third, although the guidelines and consensus documents provide direction, the issues of interoperability, quality control, and regulatory approaches still lag.

This fragmentation makes it difficult for laboratory directors and policymakers to decide where to invest. In response, this review organizes the existing evidence according to a a four-level evidence maturity gradient, from workflow efficiency and diagnostic consistency, through AI performance, to clinical outcomes and the maturity of implementation conditions.We first explore what automation has changed at the laboratory level, then examine the incremental value of AI, and finally assess whether the benefits of the laboratory are translated into benefits for patients, before identifying priorities for the next phase of evidence generation.

## 2 Methods

The literature data resources for this review were retrieved from PubMed, Web of Science, Embase and Chinese biomedical literature, from 2020 to August 2026. Representative search terms include “total laboratory automation”, “laboratory automation”, “digital microbiology”, “artificial intelligence”, “machine learning”, “turnaround time”, and “antimicrobial stewardship”. Inclusion criteria are as follows: 1) Peer-reviewed observational studies: single-center before-and-after evaluations and multi-center audits; 2) Randomized controlled trials (RCTs) and quasi-experimental studies; 3) Systematic reviews and meta-analyses, which constitute the highest-weight tier of the evidence base; 4) Clinical practice guidelines and expert consensus, each limited to its specific subject matter; 5) Methodological and validation studies; 6) Vendor-reported performance data, which are used only as background information and explicitly attributed as such. Exclusion criteria include: 1) Unvalidated single-center model development reports: admitted only as developmental-stage evidence with an explicit note that they are not prospectively validated, and never treated as clinical effectiveness evidence; 2) Literature outside clinical microbiology, or not directly related to laboratory automation and AI; 3) Grey literature: excluded, except for guidelines and consensus statements.

Studies were categorized according to the evidence maturity levels proposed in this review. These levels were assigned based on evidence type and the claim that each study could support, rather than publication type alone. At the base level, which includes workflow efficiency and diagnostic consistency, we placed TLA before-and-after studies, multi-center audits, and concordance evaluations of automated reading, identification, and susceptibility testing against manual reference standards. The second level (technical performance in image interpretation and resistance prediction) included validation studies and systematic reviews of these studies. The third level (patient outcomes) included randomized and quasi-experimental studies of rapid testing and automation, including the RAPIDO, ePlex, and FAST trials. The top level (standardization, quality control, interoperability, and regulation) includes guidelines, consensus statements, and methodological studies on interoperability (HL7/FHIR) and internal quality control and external quality assessment (EQA).

## 3 TLA in clinical microbiology: workflow efficiency and diagnostic consistency

Automated systems now cover most steps of culture-based workflow, from specimen reception to result reporting (**Figure 1**). Such a workflow map places subsequent evidence in a manner that helps clarify which step a given study addresses and where gains or gaps arise.

**Figure 1:**
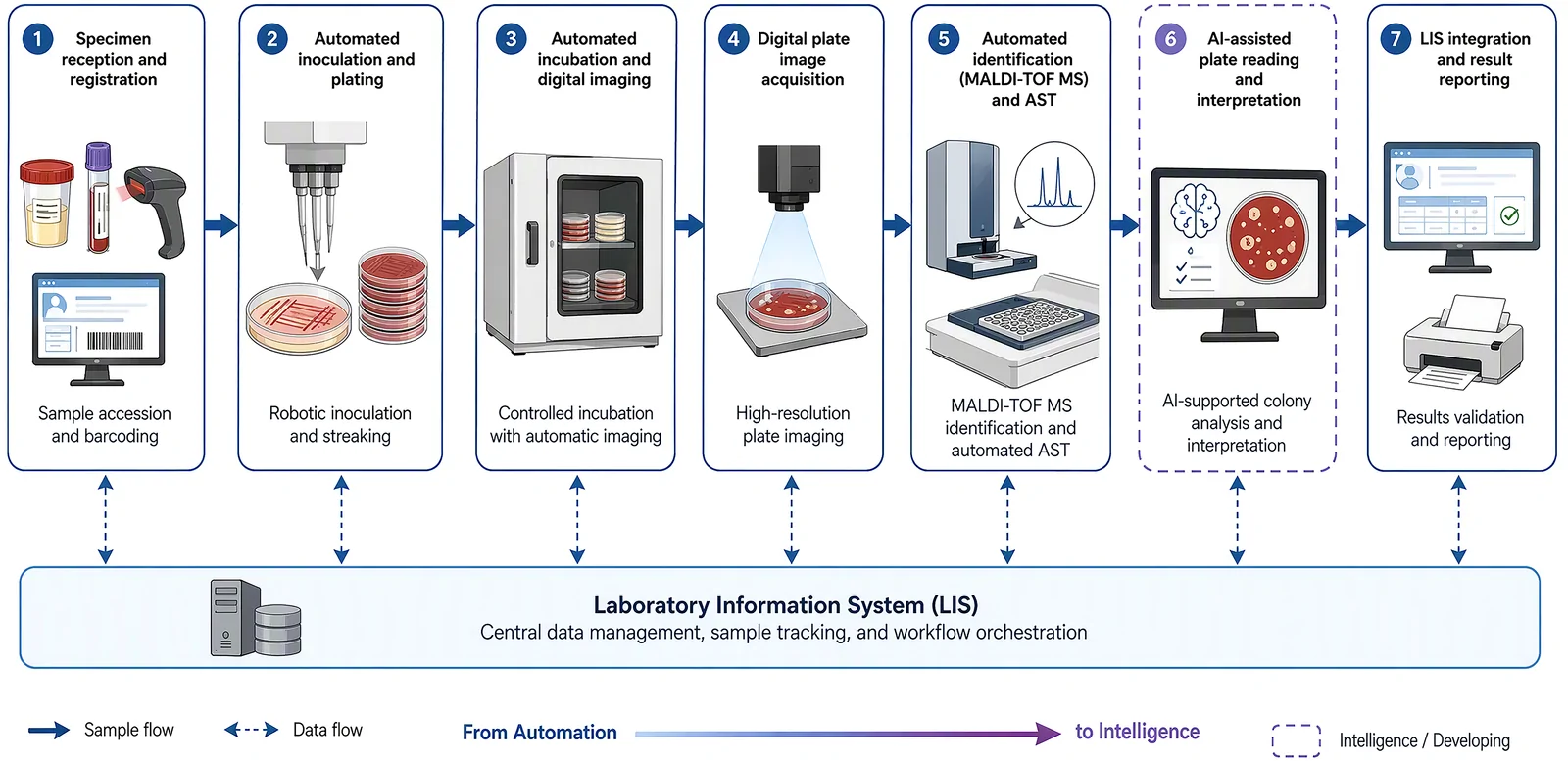
Workflow of total laboratory automation in clinical microbiology. Solid blue arrows show the physical specimen pathway from reception to reporting, including automated inoculation, incubation with digital imaging, image acquisition, identification, susceptibility testing, and result reporting through the LIS. Dashed blue arrows show the data exchange between each step and the LIS. The dashed box marks the AI-assisted plate reading and interpretation of results as a stage under development. LIS provides central data management, sample tracking, and workflow scheduling.

Beyond the analytical core, the microbiology value chain also includes pre-analytical steps (specimen acceptance and rejection, transport, and accessioning) and post-analytical steps such as result validation, critical-result communication, and integration with clinical decision support (Rhoads et al., 2014; Bailey et al., 2019). Laboratory information systems (LIS) and expert-system-based result validation are part of this wider infrastructure, and electronic reporting to clinicians and public health authorities is increasingly expected (Rhoads et al., 2014). The evidence base for these peripheral steps is more fragmented than the evidence for the analytical core: standardized, cross-institutional data on automated specimen acceptance and rejection, transport integration, and critical-result communication in microbiology remain limited, and the degree of integration with clinical decision support varies widely. We therefore focus the remainder of this section on the analytical core, while noting that gains measured there may not reach clinicians unless the surrounding pre- and post-analytical processes are addressed.

### 3.1 TAT, productivity, and workforce efficiency

In fact, the most consistent finding across automation studies is a significant reduction in cultural TAT. In a comparative study of urine cultures, Khalid et al. reported that TAT for negative cultures decreased from 49.68 to 34.38 h and for positive cultures from 50.42 to 34.62 h after the introduction of a TLA platform (Khalid et al., 2026). In addition, productivity measured as cultures per full-time equivalent (FTE) increased by 3.26-fold in the same report (Khalid et al., 2026). Also as a supporting point, a multi-center audit of four laboratories confirmed that TLA produced workflow benefits across different institutional settings, although the magnitude varied by site (Culbreath et al., 2021).

Gains in labor efficiency can exceed gains in throughput alone when TLA is combined with process redesign. For instance, in a conversion of a conventional laboratory to TLA with lean management, Trigueiro et al. reported that TAT for vaginal swabs fell from 70.3 to 48.2 h and for urine from 73.7 to 40.0 h (Trigueiro et al., 2024). Meanwhile, the daily sample volume rose from 492 to 621, and staffing decreased from 10 to 8.5 FTE, corresponding to an increase from 49 to 73 samples per FTE per day (Trigueiro et al., 2024). Importantly, automation alone accounted for only 37.2% of TAT reduction for vaginal swabs and 75.8% for urine, with the remainder attributed to lean process redesign (Trigueiro et al., 2024). This attribution is important because it shows that the magnitude of the automation benefit depends on how the surrounding workflow is reorganized.

Automation also reduces manual workload and operator variability. Alzahrani et al. reported that automated inoculation and interpretation cut pre-analytical processing time by 79% to 83% and total hands-on time by 77% to 78%, with an obvious reduction in inter-operator variability (Alzahrani et al., 2026). Urine culture and screening specimens for methicillin-resistant *Staphylococcus aureus* (MRSA), extended-spectrum beta-lactamase (ESBL) producers, and vancomycin-resistant enterococci (VRE), Cherkaoui et al. documented a shorter TAT in several specimen categories after the implementation of TLA (Cherkaoui et al., 2020).

Nonetheless, these gains were not uniform across specimen types or settings. For instance, in the study by Trigueiro et al., automation and process redesign contributed differently across specimen classes, and in the multi-center audit, benefits varied at the site level, indicating that the combination of samples, staffing, and workflow configuration can modify the magnitude of benefits (Culbreath et al., 2021). Moreover, existing evidence is primarily observational, based on before-and-after data, and cannot rule out the influence of long-term trends or concurrent quality improvement initiatives on the observed improvements. Taken together, available evidence indicates that TLA reduces TAT and manual workload in observational studies, although the scale of benefit depends on specimen type and workflow configuration, and the evidence quality remains relatively low.

### 3.2 Automatic reading, identification and antimicrobial susceptibility testing consistency

Before replacing manual reading, the automated system not only needs to be faster but also must be consistent with the reference method. Regarding concordance evidence, covering the automatic reading of culture plates, the interpretation of early culture results, and strain typing based on the entire genome. Chiu et al. compared an automatic culture plate reading system with a manual standard-care method. They compared 1519 urine culture samples and found that the difference in growth detection was 4.74% and the difference in strain identification was 3.69% (Chiu et al., 2022). Among interpretable antimicrobial susceptibility results, 0.52% contained major errors (MEs), 0.45% contained very major errors (VMEs), and 1.71% contained minor errors (mEs) (Chiu et al., 2022).

To interpret these figures, the error categories need definition. In antimicrobial susceptibility testing, a very major error is a false-susceptible result (the test reports susceptibility when the reference method reports resistance), a major error is a false-resistant result, and a minor error is a discrepancy involving an intermediate interpretation; categorical agreement is the proportion of results assigned to the same interpretive category by the test and reference methods (Festa et al., 2025). Commonly cited acceptability thresholds, based on CLSI verification criteria, require categorical agreement of at least 90%, with VME below 1.5% to 3% and ME below 3%, depending on the applied standard and organism pool (Haley et al., 2024; Festa et al., 2025). Against these thresholds, the VME of 0.45% and ME of 0.52% observed by Chiu et al. are below commonly cited acceptability limits, indicating that concordance met typical performance expectations in this single-center evaluation; the error rates nevertheless remain above zero and require confirmation in external settings.

Early reading on automated incubation platforms can shorten the time to actionable results without sacrificing detection sensitivity for key organisms. For example, using Copan WASPLab, Cherkaoui et al. showed that 18-hour reading achieved 100% sensitivity for MRSA and methicillin-susceptible *Staphylococcus aureus* (MSSA) detection, and 16-hour reading reached 100% sensitivity for ESBL producers and carbapenemase-producing Enterobacterales (CPE) (Cherkaoui et al., 2019). These results indicate that shortened incubation windows retain diagnostic performance for high-priority resistance markers, although performance in mixed specimens and for other organism groups requires further verification.

Similarly, whole-genome sequencing (WGS) adds another automation layer for strain typing. An automated WGS platform achieved 99% typing concordance (222 of 224 isolates), reduced TAT by 16 to 19 h, and lowered costs by 34% to 57% compared with conventional workflows (Siddall et al., 2025). The advantage of automated WGS technology lies in its consistency, efficiency, and cost reduction, which makes it an emerging option for epidemic investigation and strain typing. Its application depends on the size of the laboratory and infrastructure. **Table 1** summarizes the representative studies on TLA efficiency and concordance.

**Table 1:** Representative evidence on total laboratory automation: workflow efficiency and diagnostic consistency.

Study (year)	Platform or setting	Specimen or task	Key metrics	Direction
Khalid 2026 (Khalid et al., 2026)	WASPLab vs. manual	Urine cultures	Negative TAT 49.68 to 34.38 h; positive TAT 50.42 to 34.62 h; productivity 3.26-fold	Faster TAT, higher productivity
Trigueiro 2024 (Trigueiro et al., 2024)	TLA and Lean redesign	Vaginal swabs, urine	TAT 70.3 to 48.2 h (vaginal), 73.7 to 40.0 h (urine); 49 to 73 samples/FTE/day; automation alone contributed 37.2% and 75.8%	Faster TAT, lean redesign essential
Alzahrani 2026 (Alzahrani et al., 2026)	TLA vs. manual	Urine inoculation and interpretation	Pre-analytical time reduced 79% to 83%; hands-on time reduced 77% to 78%; inter-operator variability reduced	Lower workload and variability
Culbreath 2021 (Culbreath et al., 2021)	TLA, four laboratories	Mixed microbiology workflow	Workflow benefits across sites with variable magnitude	Positive, site-dependent
Cherkaoui 2020 (Cherkaoui et al., 2020)	TLA	Urine cultures; MRSA, ESBL, VRE screening	Shorter TAT across specimen classes	Faster TAT
Chiu 2022 (Chiu et al., 2022)	Automated plate reader vs manual	Urine cultures (n = 1519)	Growth difference 4.74%; identification difference 3.69%; ME 0.52%; VME 0.45%	High concordance, low error rates
Cherkaoui 2019 (Cherkaoui et al., 2019)	WASPLab early reading	MRSA/MSSA, ESBL, CPE screening	18 h sensitivity 100% (MRSA/MSSA); 16 h sensitivity 100% (ESBL/CPE)	Early reading retains sensitivity
Siddall 2025 (Siddall et al., 2025)	Automated WGS platform	Strain typing (224 isolates)	99% typing concordance; TAT reduced 16 to 19 h; cost reduced 34% to 57%	High agreement, faster, lower cost

CPE, carbapenemase-producing Enterobacterales; ESBL, extended-spectrum beta-lactamase; FTE, full-time equivalent; ME, major error; MRSA, methicillin-resistant *Staphylococcus aureus*; MSSA, methicillin-susceptible *Staphylococcus aureus*; TAT, turnaround time; TLA, total laboratory automation; VME, very major error; VRE, vancomycin-resistant enterococci; WGS, whole-genome sequencing.

Therefore, consistent evidence shows that automated reading, early interpretation, and WGS typing achieve high agreement with the reference method in terms of a common workflow and key resistance markers. At present, the error rate remains relatively low but is not zero. Moreover, most of the studies were conducted at a single institution and used manual standards as the benchmark for comparison. The benefits in terms of efficiency are in the same direction, but their magnitude depends on the sample combination and the redesign of the process. This combination of consistent direction and contextual variability sets the stage for the next question: What does AI add to an automated platform?

### 3.3 Potential disadvantages and implementation barriers of automation

Automation also carries potential disadvantages that should be weighed against the efficiency gains described above. TLA entails high initial capital investment and ongoing consumable costs, space and infrastructure requirements, and increased dependency on vendor systems for maintenance and software updates (Lippi and Da Rin, 2019). Equipment downtime concentrates risk: when an automated line fails, manual fallback may be unavailable or slower, and the laboratory’s entire workflow can be affected (Lippi and Da Rin, 2019). Automation also changes staffing needs and skill profiles and requires specific technical training for the workforce (Lippi and Da Rin, 2019). It may be less feasible or cost-effective in smaller or resource-limited laboratories (Thomson et al., 2019). Because the evidence for automation benefits is strongest in high-throughput, standardized workflows, laboratories processing heterogeneous or low-volume specimen mixes may see smaller gains and disproportionate implementation costs. These considerations argue for institution-specific cost-benefit analysis rather than universal adoption (Thomson et al., 2019).

## 4 AI in microbiology laboratories: from image reading to antimicrobial resistance prediction

### 4.1 AI-based plate reading and colony recognition

In the field of clinical microbiology, the application of AI initially focused mainly on image interpretation. Convolutional neural networks (CNNs) would classify colonies based on digital images captured by automated equipment. Kong et al. applied CNNs for microbial colony recognition and reported accuracies of 98.80% with the GoogLeNet architecture and 93.40% with AlexNet across five image categories (four colony types plus blood agar background) (Kong et al., 2025). These data indicate that, for clearly defined colony types, image classification technology has matured, although these models were trained and evaluated based on image sets from specific institutions.

The clinical value of AI depends on its integration into the reporting workflow. Davidson et al. evaluated an AI-assisted plate-reading system (PhenoMATRIX) and found that the automated reports shortened time to final result release by 1.3 hours, and further optimized the manual review by saving 5.3 hours (Davidson et al., 2026). In the same evaluation, AI-based automated reporting without an optimized human review pathway increased TAT, which demonstrates that the benefit is conditional on workflow design rather than being automatic (Davidson et al., 2026). This conditional result mirrors the lean redesign dependence observed in TLA efficiency studies and warns against treating AI reporting as an isolated intervention.

### 4.2 ML for antimicrobial susceptibility and resistance prediction

ML is employed to predict the sensitivity and resistance to antimicrobial drugs based on genomic, proteomic and phenotypic data. A comprehensive review conducted by Kim et al. summarized the current practice, limitations, and clinical perspectives of ML techniques for AMR predicting (Kim et al., 2022). This review concluded that ML models demonstrate excellent predictive performance on curated datasets, but their robustness, calibration, and generalizability across different institutions and time periods require further validation in practical applications (Kim et al., 2022). Therefore, it is necessary to have standardized data formats and conduct prospective external validation before using ML to predict antimicrobial resistance and guide individual treatment decisions. **Table 2** summarizes representative evidence of AI performance in a microbiology laboratory.

**Table 2:** Representative evidence on artificial intelligence performance in clinical microbiology.

Model or platform	Task	Performance metric	Study-level limitation
GoogLeNet CNN (Kong et al., 2025)	Microbial colony recognition (5 classes)	Accuracy 98.80%	Institution-specific image set; no external validation reported
AlexNet CNN (Kong et al., 2025)	Microbial colony recognition (5 classes)	Accuracy 93.40%	Institution-specific image set; no external validation reported
PhenoMATRIX AI-assisted reading (Davidson et al., 2026)	Automated reporting of plate results	Time to result release reduced 1.3 h; optimized review reduced a further 5.3 h	Benefit conditional on workflow; automated reporting alone increased TAT
ML for AMR prediction (review) (Kim et al., 2022)	AMR prediction	Promising performance on curated datasets	Limited real-world validation, calibration, and generalizability

AMR, antimicrobial resistance; CNN, convolutional neural network; ML, machine learning; TAT, turnaround time.

### 4.3 Limitations: data dependence, generalizability, and interpretability

The performance data above should be interpreted in light of three structural limitations. First, most models are trained and tested based on data from single laboratories, which increases the risk of domain bias when the model encounters different instruments, media, or population mixtures (Kong et al., 2025). Second, there are inconsistencies in the model calibration and reporting of decision thresholds. Even if the model achieves high accuracy on a balanced validation set, it cannot be guaranteed to perform well in the unbalanced distribution of real clinical samples (Kim et al., 2022). Third, the interpretability of deep learning models remains limited, which restricts their application in laboratories and regulatory reviews.

The conditional nature of AI benefits deserves further emphasis. Nevertheless, the evaluation of PhenoMATRIX showed that automated reporting can prolong rather than shorten TAT when human reviews are not reorganized in parallel (Davidson et al., 2026). Consequently, AI reading and resistance prediction currently function as assistive layers on automated platforms, not as autonomous replacements for laboratory staff. In other words, prospective multi-center validation, external test sets, and explicit workflow integration are required before performance metrics can be translated into operational claims.

The evidence thus indicates that AI achieves high technical performance in image classification and resistance prediction, but the clinical translation of these models is immature and their operational benefit is workflow-dependent. This raises the central question of the review: Can improved laboratory performance translate into improved clinical outcomes?

## 5 From laboratory metrics to clinical outcomes: the evidence gap

### 5.1 Randomized evidence on rapid testing and automation

RCTs provide the strongest available tests to determine whether faster diagnostics improve patient outcomes. Randomized evidence focuses on rapid molecular testing and antimicrobial susceptibility testing, whereas direct outcome data for TLA remain mostly observational. These results are notable for their overall consistency and failure to show broad clinical superiority. For instance, an early randomized trial by Bruins et al. found that shortening the TAT of microbiological procedures did not improve the clinical outcomes in hospitalized patients (Bruins et al., 2005). In accordance with this, the RAPIDO trial reached a similar conclusion: rapid microbial identification in bloodstream infection (BSI) did not improve clinical outcomes compared with routine workup (MacGowan et al., 2020). Similarly, a single-center evaluation of ePlex blood culture identification panels showed mixed results, with some process improvements but no consistent benefit on hard clinical endpoints (Caspar et al., 2024).

More recent trials have confirmed this pattern while identifying subgroups that may benefit. In the FAST randomized clinical trial, rapid antimicrobial susceptibility testing for gram-negative bacteremia shortened the time to antibiotic escalation or de-escalation by 14 h (Banerjee et al., 2026). The probability that fast testing was more desirable on the desirability of outcome ranking (DOOR) scale was 48.8% (95% confidence interval 45.3 to 52.4%), which did not meet the prespecified threshold for superiority (Banerjee et al., 2026). In the carbapenem-resistant subgroup, the time to effective therapy improved from 28 to 9.5 h (Banerjee et al., 2026). Kim et al. randomized patients with BSI undergo rapid molecular testing using BioFire BCID2 (Kim et al., 2026). The time from randomization to first effective antibiotic adjustment fell from 64.5 to 23.2 h (P < 0.001), persistent bacteremia decreased from 11.5% to 5.7%, and 30-day mortality and hospitalization were unchanged (Kim et al., 2026).

Observational data points in the same direction. In a single-centre observational study, Qin et al. reported that laboratory automation reduced the time from specimen collection to final report from 95.99 to 60.81 h and shortened turnaround for gram-negative bacteria by 44.87 h (Qin et al., 2025). However, twenty-eight-day mortality did not differ between the automated and manual periods (Qin et al., 2025). Such an observational design precludes causal interpretation of the mortality comparison, although the direction of the laboratory and clinical findings aligns with randomized evidence. **Table 3** summarizes the clinical outcome studies.

**Table 3:** Randomized and observational evidence linking rapid testing and automation to clinical outcomes.

Study (year)	Design	Intervention	Primary endpoint	Result direction
Bruins 2005 (Bruins et al., 2005)	RCT	Shortened microbiological TAT	Clinical outcomes in hospitalized patients	No improvement (negative)
RAPIDO 2020 (MacGowan et al., 2020)	RCT	Rapid microbial identification in BSI	Clinical outcomes	No improvement (negative)
Caspar 2024 (Caspar et al., 2024)	Single-centre RCT	ePlex blood culture identification panels	Clinical impact and costs	Mixed
FAST 2026 (Banerjee et al., 2026)	RCT	Fast antimicrobial susceptibility testing	DOOR at 30 days	Not superior overall (48.8%); faster escalation; carbapenem-resistant subgroup improved
Kim 2026 (BCID2) (Kim et al., 2026)	RCT	Rapid molecular BSI testing	Time to effective adjustment; hard endpoints	Process improved; mortality and hospitalization unchanged
Qin 2025 (Qin et al., 2025)	Observational, single center	Laboratory automation for blood cultures	TAT; 28-day mortality	TAT reduced; mortality unchanged

BSI, bloodstream infection; DOOR, desirability of outcome ranking; RCT, randomized controlled trial; TAT, turnaround time.

Although some randomized evidence suggests that faster diagnostics shorten the time to appropriate treatment indicators, the overall clinical superiority of hard endpoints has rarely been demonstrated. Positive signals are concentrated in defined subgroups, such as patients infected with carbapenem-resistant organisms. Nevertheless, it should be noted that this pattern requires explanation rather than dismissal, which should guide the design of future interventions.

### 5.2 Integration with AMS

One recurring explanation for the laboratory-to-outcome gap is that faster results change management only when coupled with an active AMS response. Although clinical decision support systems provide part of this coupling, the strongest evidence comes from randomized designs. In a trial by McGregor et al., a computerized clinical decision support system reduced inappropriate antimicrobial use in hospitalized patients (McGregor et al., 2006). Subsequent evidence syntheses broaden this finding to AI-based tools. Matsuo et al. summarized the prediction, clinical applications, and implementation challenges of AI in AMS (Matsuo et al., 2026), and Al Mazrouei et al. mapped the impact of AI on antimicrobial prescription, selection, resistance, and stewardship in a scoping review (Al Mazrouei et al., 2025). At the application level, AI-driven clinical decision support was evaluated against rapid susceptibility interpretation in multidrug-resistant pathogens (Lin et al., 2025).

These AMS-focused studies suggest that the laboratory benefit reaches patients mainly through a chain: faster, more accurate results, real-time communication, and action by clinicians or stewardship teams. When any link in this chain is weak, the clinical effect disappears. The available randomized trials vary in how strongly they integrate such stewardship responses, and this variation may explain part of the heterogeneity in their results.

Therefore, evidence regarding clinical outcomes therefore supports the need for a cautious and comprehensive analysis. Laboratory automation and rapid diagnostic techniques do indeed reliably improve process indicators, but their impact on mortality, hospitalization, and other hard endpoints is inconsistent. Generally, they are neutral in intention-to-treat analyses. This gap can be explained by four dimensions: endpoint selection, length of the effect chain, degree of AMS integration, and contrast in evidence grade. These dimensions also define where the implementation conditions become decisive, which is the subject of the next section.

## 6 AI-assisted evidence synthesis and implementation conditions

### 6.1 AI-assisted literature screening for evidence-based practice

Generating reliable evidence for laboratory automation has been labor-intensive for a long time. AI tools have been proposed to accelerate literature screening and evidence synthesis. A systematic review and meta-analysis by Feng et al. found that AI-based screening achieved a recall of 0.928 and a precision of 0.200 when maximizing recall and a recall of 0.708 with a precision of 0.461 when maximizing precision (Feng et al., 2022). These figures indicate that AI screening can reliably retrieve relevant records but at the cost of retrieving many irrelevant records, which means that currently, full automation of the screening step is not yet safe.

Large language models (LLMs) now appear in this space as well. A scoping review of 222 studies by Harasgama et al. found that 31.5% focused on generative pretrained transformer (GPT) models, 54.1% of the included publications appeared in 2024, and only 4.1% reported time or workload outcomes (Harasgama et al., 2026). Thus, the rapid growth in publications reflects more interest in adoption than in evidence of actual efficiency gains, and direct comparisons between traditional ML and LLM tools remain rare (Harasgama et al., 2026). A separate scoping review by Lieberum et al. concluded that LLM use for systematic reviews is increasing but not ready for routine use (Lieberum et al., 2025).

Exploratory studies in clinical microbiology support this view. In resistance mechanism detection, Giske et al. found that GPT-4-based agents achieved 81.9% accuracy compared to 94.4% for conventional methods in their test set (Giske et al., 2024). In diagnostic case scenarios, Sallam et al. reported a variable ChatGPT performance (Sallam et al., 2023). For report generation, Hu et al. showed that a pretrained ChatGPT model produced reports from automated identification and susceptibility testing systems, with quality dependent on the prompt and data structure (Hu et al., 2025). Commentary articles, such as those by Egli, argue that LLMs could reshape clinical microbiology workflows, although the structured evaluations above do not currently support this claim (Egli, 2023).

Despite these advances, the integration of AI tools with a specific evidence base for laboratory automation remains underexplored. Existing reviews that combine automation, informatics, and AI in clinical microbiology rarely make explicit the connection between AI-based evidence synthesis and the generation of evidence required by automation research. This review treats this connection as a deliberate implementation condition rather than a side observation.

### 6.2 Guidelines and expert consensus

Guidelines and consensus documents provide the most actionable direction for current practices. For instance, an expert consensus on automated workflow in clinical microbiology laboratories was jointly published by the Laboratory Medicine Branch of the Chinese Association of Medical Equipment and the Chinese Society of Laboratory Medicine (Chinese Society of Laboratory Medicine, Chinese Medical Association and Laboratory Medicine Branch of China Association of Medical Equipment, 2024). It covers the application, verification, and quality management of automated workflow. Document positions automation as an accepted operational standard while emphasizing institution-specific verification.

The American Society for Microbiology published evidence-based laboratory medicine practice guidelines for the diagnosis of BSI using rapid methods (Wolk et al., 2025). The guidelines include eight GRADE-rated recommendations and strongly recommend rapid testing combined with active communication to shorten the time to targeted therapy and reduce the length of stay while grading the evidence for mortality benefits as low (Wolk et al., 2025). This recommendation concers BSI and should not be generalized to all automated workflows.

### 6.3 Interoperability, quality control, and implementation barriers

Implementation depends on standardization, which is only partially implemented. On the quality control side, Fischer et al. described the setup and validation of an integrated internal quality control panel for monitoring TLA processes (Fischer et al., 2026). Scherz et al. proposed a simple approach to internal quality assurance in diagnostic microbiology (Scherz et al., 2017). These methodological contributions demonstrate feasible solutions, but no unified quality control and EQA framework for automation and AI systems currently exists. The regulatory pathways for AI-based laboratory software remain unclear. The general landscape of AI-based medical devices was reviewed for the United States, although specific approvals for microbiology AI products could not be confirmed from available evidence (Benjamens et al., 2020).

Interoperability between instruments, LIS, and clinical systems is another barrier. Based on the evidence examined, we were unable to confirm whether the data exchange standards in the HL7 and FHIR families had been consistently applied to microbiology automation and AI outputs across vendors and institutions. In the absence of shared data standards, multi-center validation of AI models and benchmarking of automated workflows remain difficult to compare.

Taken together, the implementation layer provides direction through guidelines and consensus, but it lacks the standardized quality control, interoperability, and regulatory scaffolding needed to scale automation and AI across institutions. The combination of this direction guidance and the structural deficiencies mentioned in this review is expected to be a crucial step toward achieving full-process automation in microbiological laboratories.

## 7 Discussion and conclusions

### 7.1 Comprehensive analysis along the evidence maturity ladder

As shown in **Figure 2**, the evidence assembled in this review forms a consistent maturity gradient. Workflow efficiency and diagnostic consistency showed the most mature evidence at the base of the ladder. Multiple observational studies and multi-center audits document shorter TAT, higher productivity, and acceptable concordance with manual standards. The evidence grade was low and the effect sizes varied with the specimen mix and workflow design. In the second tier, AI performance evidence for plate reading and resistance prediction is abundant and technically strong; however, clinical translation remains immature, and operational benefits are conditional on workflow integration. In the third tier, patient-oriented evidence is the least mature among clinical evidence types: randomized trials consistently improve process metrics but rarely demonstrate overall superiority in hard clinical outcomes, with positive signals concentrated in defined subgroups. At the top of the ladder, standardization, quality control, and regulatory layers are still under development, which limits the extent to which local gains can scale.

**Figure 2:**
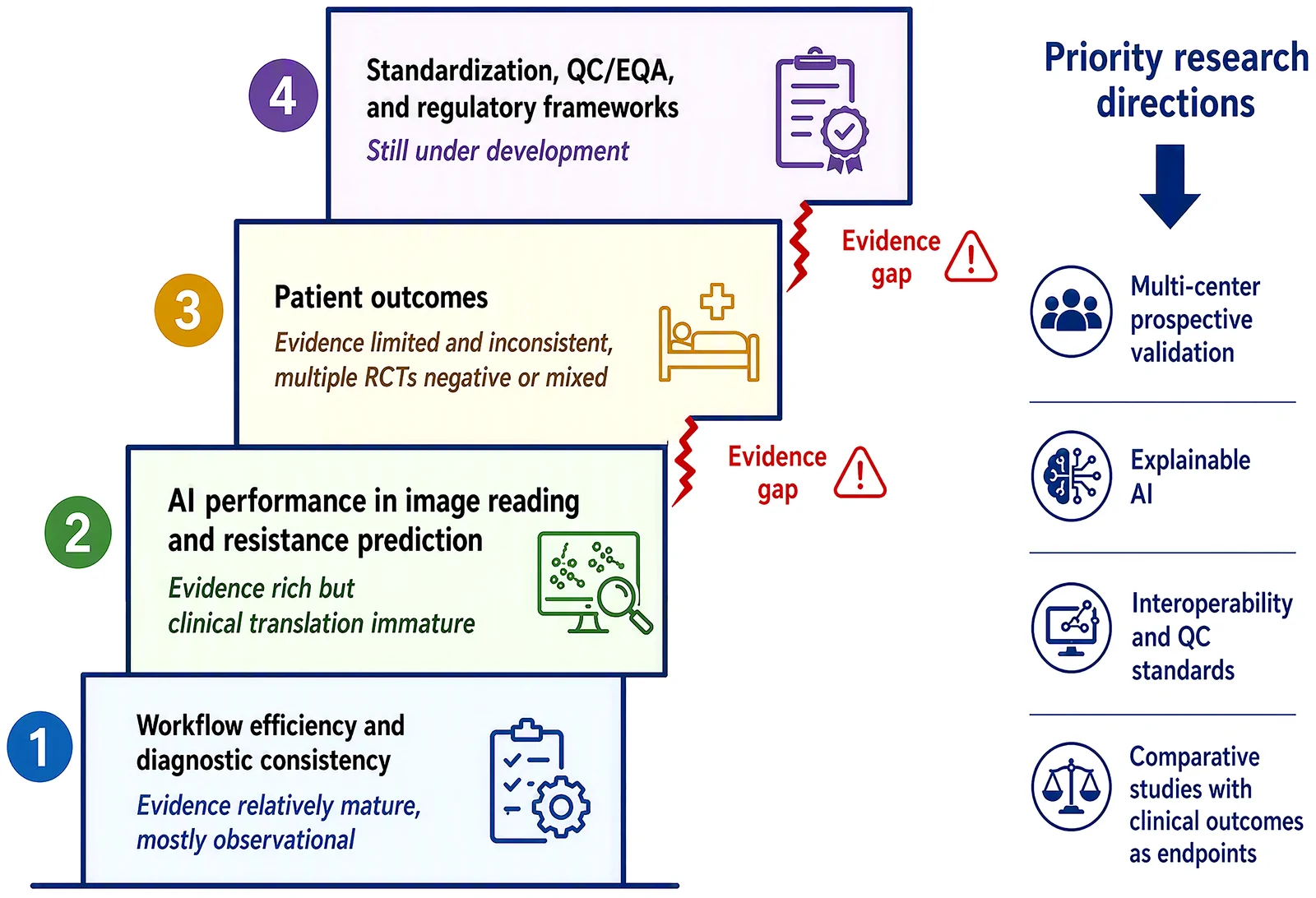
Evidence maturity ladder for automation and AI in clinical microbiology. From bottom to top: Workflow efficiency and diagnostic consistency (relatively mature, mostly observational); AI performance in reading and resistance prediction (abundant, immature for clinical translation); patient outcomes (limited, inconsistent); and standardization, quality control, and regulatory frameworks (under development). Breaks between layers indicate gaps in evidence. Arrows on the right list the priority directions: multi-center prospective validation, explainable AI, interoperability and quality control standards, and comparative studies with clinical outcomes as endpoints.

These four dimensions explain why laboratory gains do not automatically become patient gains. First, endpoint selection matters: efficiency and intermediate endpoints improve more readily than hard outcomes, such as mortality. Second, the effect chain from laboratory results through clinician decisions to patient outcomes is long, and each step can dissipate the benefit. Third, the degree of AMS integration may determine whether faster results change the therapy, and trials with stronger stewardship coupling may show clearer process gains. Fourth, the evidence grade differs sharply between observational efficiency studies and randomized outcome trials; therefore, the two bodies of evidence should not be read as comparable in strength.

The conclusions of this review are that available evidence explains that TLA reduces TAT and manual workload, though the scale of benefit depends on specimen type and workflow configuration; that AI achieves high technical performance in assistive tasks; and that patient-level benefits remain unproven overall. The contested area is not whether these technologies work on the bench, but how large the clinical effect is and under which conditions it appears.

### 7.2 Distinguish among TLA, AI, and related approaches

**Table 4** distinguishes six related technologies or approaches - TLA, ML, AI-assisted image interpretation, rapid molecular testing, AMR prediction, and AMS integration - according to their core function, representative evidence base, and current role and evidence maturity.

**Table 4:** Distinguishing TLA, AI, and related approaches in the clinical microbiology laboratory.

Technology or approach	Core function	Representative evidence base	Current role and evidence maturity
TLA	Physical automation of specimen handling, inoculation, incubation, digital imaging, and result documentation	Observational before-after studies and multi-center audits (Culbreath et al., 2021; Khalid et al., 2026)	Established operational standard; reduces TAT and manual workload, with context-dependent magnitude
ML	Algorithms trained on data for classification or prediction, e.g., AMR prediction	Institution-built datasets and reviews (Kim et al., 2022)	Assistive; requires standardized data and prospective external validation
AI-assisted image interpretation	Deep learning (e.g., CNNs) for colony recognition and plate reading	Single-center evaluations (Kong et al., 2025; Davidson et al., 2026)	Assistive reading; benefit conditional on workflow integration
Rapid molecular testing	Nucleic acid amplification or syndromic panels for direct pathogen detection	Randomized trials (Bruins et al., 2005; MacGowan et al., 2020; Kim et al., 2026)	Process improvements; inconsistent hard-endpoint benefit
AMR prediction	ML models predicting resistance from genomic, proteomic, or phenotypic data	Reviews and curated-dataset studies (Kim et al., 2022)	Research/assistive; not yet routine for individual treatment decisions
AMS integration	Decision support coupling laboratory results to therapy decisions	Randomized trials and scoping reviews (McGregor et al., 2006; Al Mazrouei et al., 2025)	Key link translating laboratory results into clinical benefit

TLA, total laboratory automation; TAT, turnaround time; ML, machine learning; AMR, antimicrobial resistance; AI, artificial intelligence; AMS, antimicrobial stewardship; CNN, convolutional neural network.

### 7.3 Limitations

This study has several limitations. 1) Most efficiency and concordance data come from single institutions with before-and-after designs, which limits causal inference and generalizability. 2) Regulatory status and the existence of dedicated standardization documents could not be confirmed for several jurisdictions; therefore, the review reports only what the verified evidence supports. 3) AI performance data are largely derived from institution-built datasets without external validation, and this review treats them as technical performance evidence rather than clinical effectiveness evidence. 4) The review did not rely on analogy-based arguments from other clinical fields as primary evidence.

### 7.4 Future directions and testable questions

The priority for the next phase is not an additional single-center performance validation. Four directions follow directly from the maturity gradient, matching the priority list shown in **Figure 2**. First, multi-center prospective validation with standardized endpoints is needed for both automation and AI systems, including external test sets and pre-specified workflow integration. Second, explainable AI and decision-support design need systematic evaluation, because clinician acceptance and auditability determine whether high technical performance translates into routine use. Third, standardization must move beyond guidelines toward interoperable data formats, unified quality control and EQA approaches, and clearer regulatory pathways, because these determine whether local success can scale. Fourth, comparative studies with clinical outcomes as endpoints, designed around AMS integration and pre-specified subgroups, are required to identify which patient groups benefit the most.

These directions can be formulated as testable questions: Does AI-assisted reading improve patient outcomes when embedded in a defined reporting and stewardship workflow? Do automated systems reduce the time to effective therapy for carbapenem-resistant infections beyond the process metric? Can interoperable data standards enable external validation of resistance prediction models across institutions? Does adding explainability tools change clinician acceptance and regulatory reviews of AI-based laboratory software? Can unified quality control and EQA schemes be validated across automated platforms? AI-assisted evidence tools, once their own performance is better characterized, can accelerate the synthesis needed to answer such questions, provided that screening precision and outcome reporting improve simultaneously.

The evidence maturity gradient thus provides both a map of the current field and a prioritization rule. Investment in automation and AI should follow the gradient: consolidate mature efficiency evidence with standardized measurement, close the validation gap for AI performance, and build clinical outcome evidence and standardization frameworks that currently lag behind the technology.
